# LONG-TERM CARE IV A REVIEW OF THE LONG-TERM CARE INSURANCE PROGRAMS IN 15 PILOT CITIES IN CHINA

**DOI:** 10.1093/geroni/igz038.2948

**Published:** 2019-11-08

**Authors:** Yan Lin, Edward A Miller, Marc A Cohen, Pamela Nadash, Peng Du

**Affiliations:** 1 Department of Gerontology, University of Massachusetts, Boston, Boston, Massachusetts, United States; 2 University of Massachusetts Boston, Boston, Massachusetts, United States; 3 University of Massachusetts Boston Gerontology Institute, LeadingAge LTSS Center @UMass Boston, Boston, Massachusetts, United States; 4 Institute of Gerontology, Renmin University of China, Beijing, China

## Abstract

China is now making all the efforts to solve the problem of who pays the bill for the rapidly increasing long-term care services. Since 2016, 15 cities in China have begun their pilot programs in long-term care insurance. Each city designed its own program. Some cities finance their long-term care services from medical insurance funding solely. Others supplement it with individual and/or employer contributions. This study documents the nature and extent of implementation of long-term care insurance across the 15 pilot cities to draw lessons for subsequent implementation nationally. This study used qualitative methods, including document review and key informant interviews with long-term care insurance administrators, medical insurance administrators, service providers in different settings and families and individuals who use long-term care services. Results reveal considerable variation in the specific attributes of the long-term care insurance programs implemented across the 15 participating cities, with respect to such characteristics as the target population, policy coverage, and payment methods, etc. Results also shed light on the achievements and challenges in implementing the long-term care insurance program. This study’s examination of long-term care insurance adoption in 15 Chinese cities serves as an important base with which to inform future national long-term care insurance design and implementation. It suggests that successful adoption of long-term care insurance across China will require administering jurisdictions to anticipate and address policy bottlenecks and implementation barriers that might otherwise inhibit program impact and effectiveness in addressing the growing long-term care needs of China’s aging population.

